# A randomized controlled trial of remote microphone listening devices to treat auditory deficits in children with neurofibromatosis type 1

**DOI:** 10.1007/s10072-022-06203-8

**Published:** 2022-06-20

**Authors:** Gary Rance, Alice Maier, Julien Zanin, Kristina M. Haebich, Kathryn N. North, Francesca Orsini, Gabriel Dabscheck, Martin B. Delatycki, Jonathan M. Payne

**Affiliations:** 1 Department of Audiology and Speech Pathology, The University of Melbourne, 550 Swanston Street, VIC 3010 Parkville, Carlton, Australia; 2 Murdoch Children’s Research Institute, Parkville, VIC Australia; 3 The Royal Children’s Hospital, Parkville, VIC Australia; 4 Department of Paediatrics, Faculty of Medicine, Dentistry and Health Sciences, University of Melbourne, Parkville, VIC Australia; 5 Victorian Clinical Genetics Services, The Royal Children’s Hospital, Parkville Victoria, Australia

**Keywords:** Neurofibromatosis type 1; Auditory; Remote-microphone device

## Abstract

**Background:**

A high proportion of patients with neurofibromatosis type 1 (NF1) present with functional hearing deficiency as a result of neural abnormality in the late auditory brainstem.

**Methods:**

In this randomized, two-period crossover study, we investigated the hypothesis that remote-microphone listening devices can ameliorate hearing and communication deficits in affected school-aged children (7–17 years). Speech perception ability in background noise was evaluated in device-active and inactive conditions using the CNC-word test. Participants were then randomized to one of two treatment sequences: (1) inactive device for two weeks (placebo), followed by active device use for two weeks, or (2) active device for 2 weeks, followed by inactive device for 2 weeks. Listening and communication ratings (LIFE-R Questionnaire) were obtained at baseline and at the end of each treatment phase.

**Results:**

Each participant demonstrated functional hearing benefits with remote-microphone use. All showed a speech perception in noise increase when the device was activated with a mean phoneme-score difference of 16.4% (*p* < 0.001) and reported improved listening/communication abilities in the school classroom (mean difference: 23.4%; *p* = 0.017).

**Discussion:**

Conventional hearing aids are typically ineffective as a treatment for auditory neural dysfunction, making sounds louder, but not clearer for affected individuals. In this study, we demonstrate that remote-microphone technologies are acceptable/tolerable in pediatric patients with NF1 and can ameliorate their hearing deficits.

**Conclusion:**

Remote-microphone listening systems offer a viable treatment option for children with auditory deficits associated with NF1.

Neurofibromatosis type 1 (NF1) occurs as a result of loss-of-function mutations within the *NF1* gene [1]. Although characterized by cutaneous, skeletal, and neoplastic abnormalities [2], the most common complications are cognitive deficits and neurodevelopmental disorders including attention deficit hyperactivity disorder (ADHD; 40–50%) and autism spectrum disorder (ASD; 25%) [3, 4]. In our recent study, a high proportion (32%) of NF1 participants also presented with significant functional hearing deficits associated with neural disruption between the cochlear nucleus and lateral lemniscus [5]. 

Management of auditory neural deficit is challenging as perception is limited, not by audibility, but by disruption of neural firing patterns. This neural distortion affects discrimination of the subtle temporal cues that distinguish complex signals (such as speech) and disrupts sound localization reducing the listener’s capacity to spatially separate sound sources to improve speech understanding in background noise [6]. Conventional hearing aids are typically of little benefit, making sounds louder, but not clearer for affected individuals [7].

In this study, we investigated the hypothesis that remote-microphone listening devices can ameliorate hearing deficits in children with NF1. These devices improve perception not by amplifying sound, but by improving the signal-to-noise ratio (SNR), i.e., level of the target signal relative to the background noise. This is achieved by recording the speaker’s voice near the mouth and digitally transmitting the signal directly to the listener’s ear. Such devices have proven useful in other populations with auditory neural deficit, including children with Friedreich ataxia [8], ASD [9], and ADHD [10].

Experimental protocols were approved by the Royal Children’s Hospital (RCH) Ethics Committee (2019.010) and prospectively registered through the Australian/New Zealand Clinical Trials Registry (ACTRN12619000525189). Patients were recruited consecutively from the RCH Neurofibromatosis Clinic as part of a NF1-phenotype study [5].

Eleven of 24 children from our previous study [5] showed clinically abnormal functional hearing (speech perception-in-noise scores outside age-based norms) and 10 of these consented to participate in the device trial (Fig. 1A). Mean Full Scale IQ of the randomized cohort was 85.8 (SD = 9.2; no participant had an intellectual disability), and 5/10 (50%) had a diagnosis of ADHD. Mean difference between “speech reception thresholds” for the study and control groups was 5.3 dB (SD = 4.0), indicating that the NF1 participants required significantly lower levels of background noise to hear/understand speech as well as their neurotypical peers. In a typical school classroom, a noise sensitivity difference of this order would correspond to a 15–20% loss of intelligibility [11] and a greatly increased risk of disengagement from learning activity [12].

**Fig. 1:**
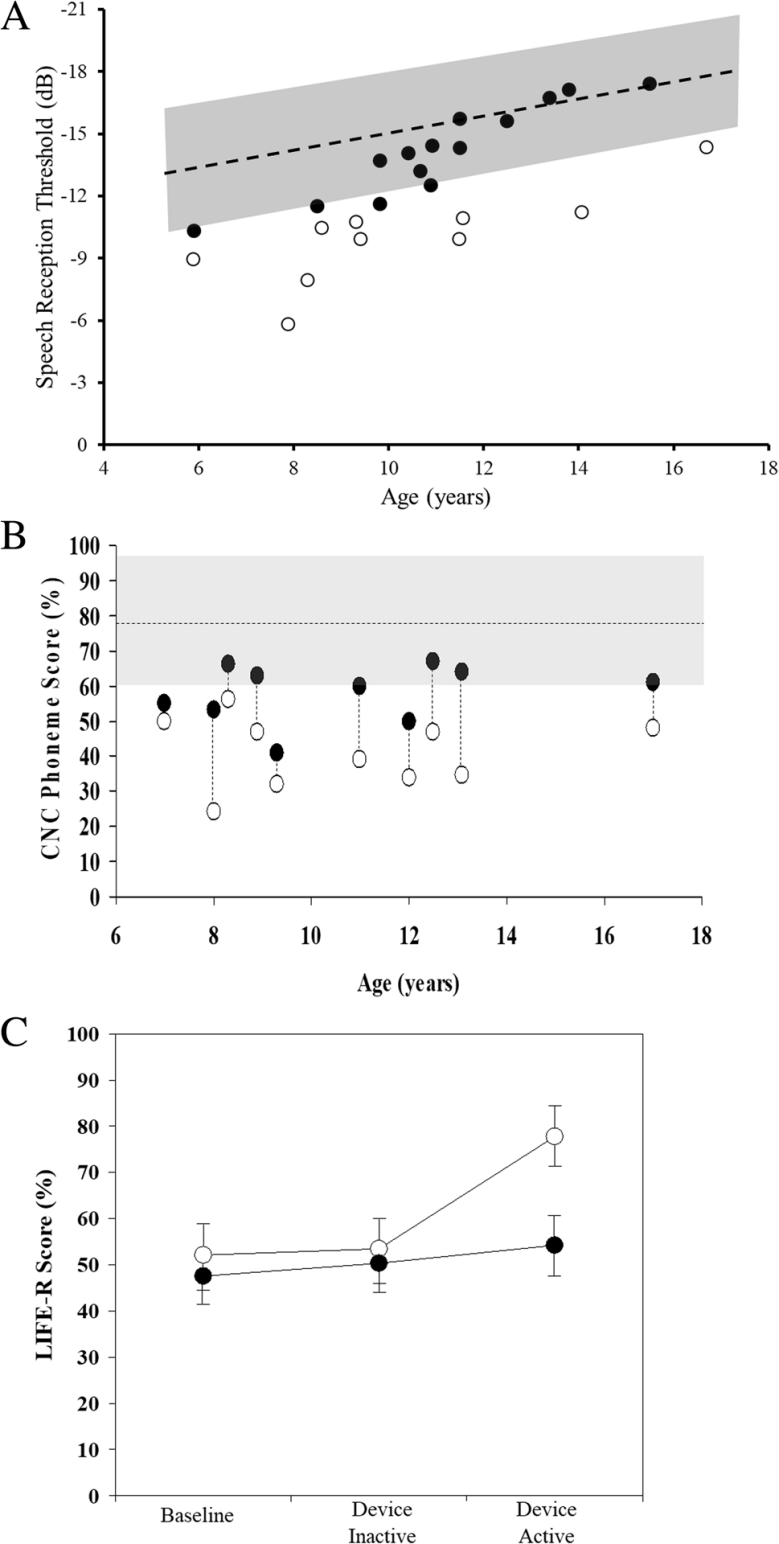
**A** Binaural speech perception in noise scores for children with NF1 [5]. Shown are speech reception thresholds for the Listening in Spatialized Noise test (DV90 condition). The shaded area represents the mean ± 2SD performance range for normally developing children. Unfilled data points are findings for participants recruited to the intervention study. **B** Open-set speech perception in noise (0 dB SNR) scores as a function of participant age. Unfilled data points show the phoneme score for each individual in the inactive device condition and the filled points are for the active device condition. The shaded area represents the (no device) 95% performance range for children with no auditory processing deficits based on published normative findings [8, 9, 13]. **C** Mean LIFE-R rating scores for intervention study participants (open data points) and classroom-teachers (filled data points) at each of the three data collection points. Error bars represent ± 1 standard error

To determine whether auditory deficits in children with NF1 are amenable to treatment, we conducted a randomized, blinded, two-period crossover study. Each participant was provided with a listening-system which consisted of a Roger-Focus receiver worn at ear-level by the child, paired with a Roger-Touchscreen microphone worn at lapel-level by the classroom teacher. Each child wore the device in school for 4 weeks. Participants were randomized to one of two treatment sequences: (1) active device for 2 weeks followed by inactive device (i.e., control) for 2 weeks or (2) inactive device for 2 weeks, followed by active device for two weeks (Fig. 2). Devices were “inactivated” by unpairing the microphone and receiver so that no signal was transmitted — despite the system appearing outwardly functional. Other than the study-audiologist, all investigators and participants were blinded to allocated sequence.

**Fig. 2:**
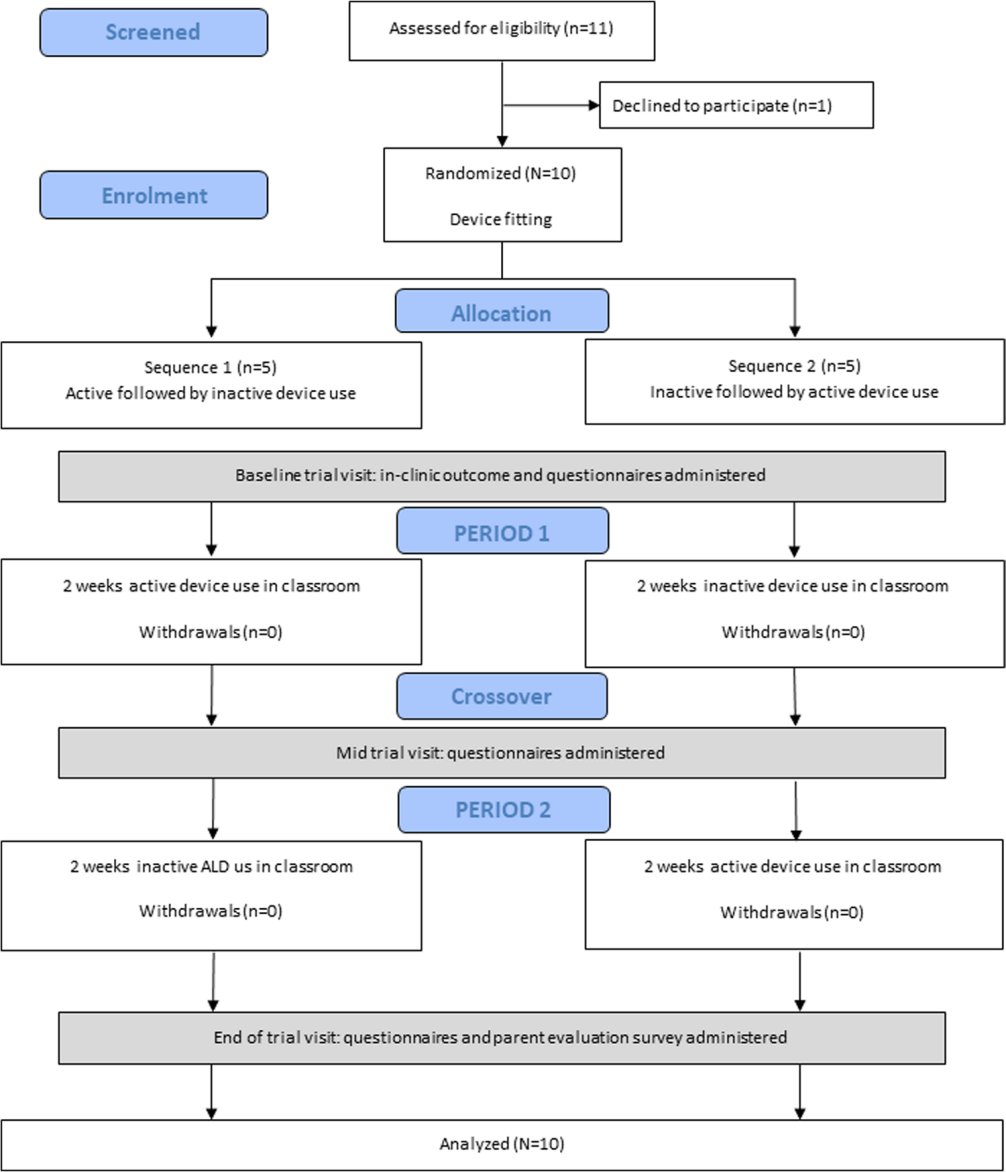
CONSORT flow diagram outlining the phases of the remote-microphone device trial

Study participants maintained good device usage throughout the study period. There were no withdrawals, and each child wore the device for at least 75% of classroom activities across the trial.

## Speech perception

At the baseline (clinic) visit, the effect of the device on speech perception-in-noise was evaluated using the Consonant-Nucleus-Consonant (CNC) Word test as per Rance et al. [8]. A speech-to-noise ratio of 0 dB was selected to replicate listening conditions in a standard classroom [13, 14]. Testing was carried out in both device-active and inactive conditions with order randomized.

Use of the remote-microphone system improved speech perception for all participants. Where mean CNC-phoneme score for the inactive-device condition was 41.4% (SD = 10.2), mean speech score for the device-activated condition increased to 57.8% (SD = 8.3), (mean difference = 16.4%, 95%CI 10.1 to 22.8%, *p* < 0.001) (Fig. 1B). Multivariate regression analyses revealed that while test order was a factor (*p* = 0.007), significantly higher CNC-phoneme scores were obtained in the device-active condition after controlling for order-effects (*p* < 0.001).

## Classroom listening

Listening and communication outcomes were assessed at baseline and at the end of each 2-week intervention period. These were evaluated using the Listening Inventory for Education-Revised (LIFE-R) questionnaire, a child- and teacher-completed measure that assesses the participant’s listening performance across 15 school-based scenarios.

Teachers were unable to discern differences in classroom listening between device conditions. Mean Teacher LIFE-R scores for the inactive-device condition were 51.4% (SD = 13.6), compared with 54.2% (SD = 15.6) for the activated condition (MD = 2.9%, 95%CI − 3.13 to 8.8, *p* = 0.30) (Fig. 1C). Regression analysis showed no treatment (*p* = 0.33) or order (*p* = 0.20) effect on the teacher ratings.

Conversely, study participants identified significant improvement in their classroom listening during the device-active condition. Mean Student LIFE-R scores for the inactive condition were 54.6% (SD = 28.8) versus 78.0% (SD = 22.5) for the activated condition (MD = 23.4%, 95% CI 5.7 to 41.2, *p* = 0.017) (Fig. 1C). Treatment sequence also showed a significant effect on outcome (*p* = 0.01) with participants rating their listening ability as considerably reduced in the inactive condition if it followed the active condition.

In summary, remote-microphone listening systems provided significant perceptual benefits with each participant showing superior speech perception scores and 6/10 children, in fact, improving to within the “normal” performance range. Importantly, these improvements translated to clear hearing benefits. Compared to the inactive condition, children with NF1 reported better classroom listening and communication when the device was activated. Teachers were not able to identify listening behavior differences, but this was likely the result of the short treatment period.

This intervention study represents the controlled implementation of device use in a mainstream-classroom context. There are however several limitations. While the effect sizes were large and robust, the trial involved reasonably small participant numbers and was conducted over a relatively short time. Larger studies trialing devices for extended periods are required to assess the longer-term impact of auditory intervention on more distal areas of functioning including learning, social, and behavioral outcomes — all common areas of concern in children with NF1 [3, 4, 12].
